# Genome-wide characterization and evolutionary analysis of heat shock transcription factors (HSFs) to reveal their potential role under abiotic stresses in radish (*Raphanus sativus* L.)

**DOI:** 10.1186/s12864-019-6121-3

**Published:** 2019-10-24

**Authors:** Mingjia Tang, Liang Xu, Yan Wang, Wanwan Cheng, Xiaobo Luo, Yang Xie, Lianxue Fan, Liwang Liu

**Affiliations:** National Key Laboratory of Crop Genetics and Germplasm Enhancement, Key Laboratory of Horticultural Crop Biology and Genetic Improvement (East China) of MOA, College of Horticulture, Nanjing Agricultural University, Nanjing, 210095 People’s Republic of China

**Keywords:** Abiotic stress, Heat shock transcription factors (HSFs), Radish, Reverse-transcription quantitative PCR (RT-qPCR)

## Abstract

**Background:**

Abiotic stresses due to climate change pose a great threat to crop production. Heat shock transcription factors (HSFs) are vital regulators that play key roles in protecting plants against various abiotic stresses. Therefore, the identification and characterization of HSFs is imperative to dissect the mechanism responsible for plant stress responses. Although the HSF gene family has been extensively studied in several plant species, its characterization, evolutionary history and expression patterns in the radish (*Raphanus sativus* L.) remain limited.

**Results:**

In this study, 33 RsHSF genes were obtained from the radish genome, which were classified into three main groups based on HSF protein domain structure. Chromosomal localization analysis revealed that 28 of 33 RsHSF genes were located on nine chromosomes, and 10 duplicated RsHSF genes were grouped into eight gene pairs by whole genome duplication (WGD). Moreover, there were 23 or 9 pairs of orthologous HSFs were identified between radish and *Arabidopsis* or rice, respectively. Comparative analysis revealed a close relationship among radish, Chinese cabbage and *Arabidopsis*. RNA-seq data showed that eight RsHSF genes including *RsHSF-03*, were highly expressed in the leaf, root, cortex, cambium and xylem, indicating that these genes might be involved in plant growth and development. Further, quantitative real-time polymerase chain reaction (RT-qPCR) indicated that the expression patterns of 12 RsHSF genes varied upon exposure to different abiotic stresses including heat, salt, and heavy metals. These results indicated that the RsHSFs may be involved in abiotic stress response.

**Conclusions:**

These results could provide fundamental insights into the characteristics and evolution of the HSF family and facilitate further dissection of the molecular mechanism responsible for radish abiotic stress responses.

## Background

Many inevitable environmental factors (e.g., heat, drought, flooding, salinity and heavy metals) trigger abiotic stress, affect plant growth and consequently increase crop losses [1–3]. Heat stress (HS), one of the major abiotic stresses, severely inhibits crop growth and development. These effects have had devastating economic impacts on the yield and quality of rice, wheat, maize and vegetable crops [4–6]. Heat shock transcription factors (HSFs) are important regulators that could contribute to controlling the differential expression of heat shock proteins (HSPs) and other functional genes in the process of protecting plants from heat and other stresses including chilling, salinity, drought and heavy metal [7, 8]. It is imperative to clarify the molecular mechanisms that govern how plants respond and adapt to HS.

Plants have developed multiple defense mechanisms and strategies to cope with adverse conditions and respond accordingly [9–11]. Under abiotic stresses, the induction of myriad proteins, including transcription factors (TFs), could regulate the expression of specific functional genes to enhance plant resistance through signal transduction pathways. Reactive oxygen species (ROS)-scavenging enzymes and HSPs are important functional proteins induced by HS, and their corresponding genes are targets of several HS-responsive TFs [12–14]. Previous studies indicate that *HSFA6b* vitally acts as a downstream regulator of the ABA-mediated heat stress response (HSR) in *Arabidopsis thaliana* [15]. Additionally, in the absence of HS, HSP genes expression is induced by the overexpression of constitutively active HsfA1d without temperature-dependent repression (TDR) domain, thus conferring effective thermotolerance in *Arabidopsis* [16]. In the tomato, cadmium (Cd) tolerance is conferred by HsfA1a through activating *COMT1* gene transcription, which partially upregulates HSP expression by inducing melatonin in accumulation [8].

In plants, the HSFs have a conserved modular structure with considerable size and sequence variability [9, 17–19]. For example, all HSFs have an N-terminal DNA-binding domain (DBD) and an adjacent bipartite oligomerization domain (HR-A/B), while only a proportion contain a nuclear localization signal (NLS) domain, nuclear export signal (NES) domain and C-terminal activator domain (CTAD) [9]. According to the peculiarities of the adjacent hydrophobic amino acid residues (HR-A/B), plant HSFs can be divided into three types, class A, B and C [20–22]. Numerous HSF families have been isolated from a range of plant species, including Chinese cabbage (*Brassica.rapa* ssp*. pekinensis*) [23], *Arabidopsis* [24], carrot (*Daucus carota*) [13], peanut (*Arachis hypogaea*) [7], rice (*Oryza sativa*) [25], maize (*Zea mays*) [26], Chinese white pear (*Pyrus bretschneideri*) [27], poplar (*Populus trichocarpa*) and barrelclover (*Medicago truncatula*) [28]. However, the comprehensive characteristics of HSF genes remains unknown in root vegetable crops, including radish.

Radish (*Raphanus sativus* L., 2n = 18), belonging to the Brassicaceae family, is one of the most economically important annual or biennial root vegetable crops [29]. Previous studies showed that 26 known and 19 novel microRNAs (miRNAs) are differentially expressed under HS in radish roots [4]. Moreover, there are several differentially expressed genes (DEGs) involved in heat stress response process in radish [30]. Recently, the available radish genome sequence provided a useful resource for whole-genome identification of TF families [31]. Nevertheless, little information on systematic characterization of HSF genes and their families in response to abiotic stresses is available in radish. This study aimed to to isolate the full-length RsHSF sequences from the radish genome, map the RsHSFs onto chromosomes and explore their expression in response to abiotic stresses. Moreover, RsHSF orthologs and paralogs were obtained and the expression pattern of RsHSFs in different radish tissues was investigated. The outcomes of this study provide an opportunity to further explore the roles of HSF genes involved in abiotic stresses and present the expansion and evolutionary history of the HSF gene family in radish.

## Results

### Identification and characterization of radish HSF proteins

To comprehensively identify the candidate HSF proteins in radish, a profile Hidden Markov Model (HMM) [32] search against NODAI radish genome protein sequences with the HSF domain (Pfam: PF00447) was performed. A total of 55 putative RsHSF gene sequences were obtained from the whole genome. SMART (http://smart.embl-heidelberg.de/) and Pfam (http://pfam.xfam.org/) were used to remove proteins with incomplete hsf-type DBD domain, while MARCOIL (http://toolkit.tuebingen.mpg.de/marcoil) was used to confirm OD (HR-A/B) domain presence. After removing sequences that encoded proteins without the complete DBD, OD (HR-A/B) and/or start/stop codons, 33 RsHSF genes remained for further analysis (Additional file 1: Table S1–S3). Subsequently, the conserved domains of all retained genes were identified by Heatster. The DBD, which consists of approximately 100 amino acids (AAs) at the N-terminus, was the most conserved domain. Overall, 31 AAs of the DBD domain were highly conserved among all RsHSFs (Additional file 2: Figure S1). NLS and NES are crucial for HSF intracellular distribution between the nucleus and cytoplasm. All RsHSFs had an NES, whereas only 13 contained the NLS (Additional file 3: Table S4).

Furthermore, the physical and chemical properties of 33 RsHSF proteins were analyzed with ExPASy (Additional file 3: Table S4). Among these, the RsHSF protein sizes varied from 238 to 2427 AAs with molecular weights (MWs) from 27.80 to 274.05 KDa, respectively. Additionally, the theoretical pI of most RsHSF proteins was < 7, with the exception *RsHSF-04*, *RsHSF-05*, *RsHSF-06* and *RsHSF-25*. All RsHSFs were classified as unstable proteins according to the instability index. The aliphatic index may be a positive factor that increased globular protein thermostability, and *RsHSF-25* had the largest aliphatic index (77.14). Most RsHSFs showed similar physical and chemical properties, while different AA sequences in non-conserved regions may alter some of the molecular characteristics.

### Intron–exon structure and conserved motif distribution

To obtain information about the diversity of RsHSF gene structure, the full length of complementary DNA (cDNA) sequences were compared with the corresponding genomic DNA sequences through Gene Structure Display Server (GSDS). To further analyze RsHSF protein motif distribution, whole sequences were subjected to the MEME web server and a total of 25 AA motifs were generated (Additional file 2: Figure.S2).

**Fig. 1:**
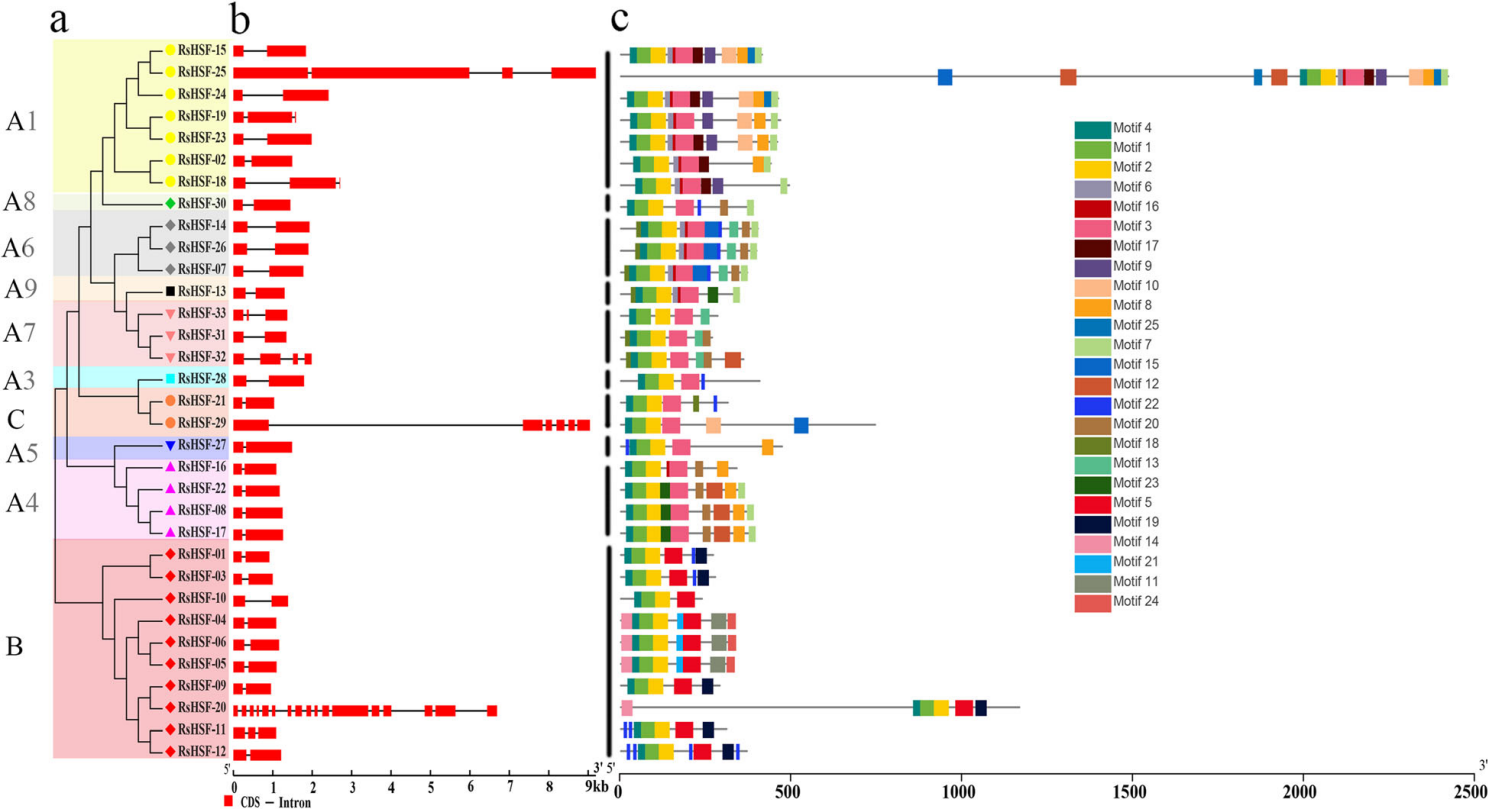
Gene structures and protein motifs of HSF family. **a** The neighborjoining phylogenetic tree of RsHSFs. **b** The exon/intron structures of RsHSF genes. The relative position is proportionally displayed based on the kilobase scale at the bottom of the figure. Red boxes and gray lines represent exons and introns, respectively. **c** The conserved motifs in radish HSFs. Different motifs and their relative positions are represented by the colored boxes

Overall, 25 of 33 RsHSF genes had one intron, while the ramaining members have two or more introns. Additionally, proteins within the same subgroups had similar motif structures. All RsHSF proteins harbored motifs 1, 2 and 4, all of which highly correspond to the conserved DBD. Moreover, *RsHSF-07*, *RsHSF-14* and *RsHSF-26* in subgroup A6 presented a similar gene and motif structure. However, some motifs were only detected in specific RsHSF protein classes. For example, motif 3 was identified in classes A and C, while motifs 9 and 17 only existed in class A1. Intriguingly, the motifs 5 and 19 were only identified in class B. A proportion of RsHSF proteins in the same class exhibited similar motif distribution, indicating that these proteins might have conserved functions (Fig. 1).

### Comparative and phylogenetic analysis of RsHSF genes

To investigate the distribution of HSF subfamilies and the evolutionary relationship among different species, 33 RsHSFs combined with 35 BrHSFs, 19 VvHSFs*,* 25 OsHSFs and 21 AtHSFs identified from Chinese cabbage (*B. rapa* ssp*. pekinensis*) [33], grape (*V. vinifera*) (https://phytozome.jgi.doe.gov/pz/portal.html) [34, 35], rice (*O. sativa*) (http://rice.plantbiology.msu.edu/) [36] and *Arabidopsis* (http://www.arabidopsis.org/) [37] were employed for comparative analysis (Additional file 4: Table S5). Based on the number of AA residues between the A and B portions of the HR-A/B region, RsHSFs were classified into three classes, namely A, B and C (Fig. 1).

**Fig. 2:**
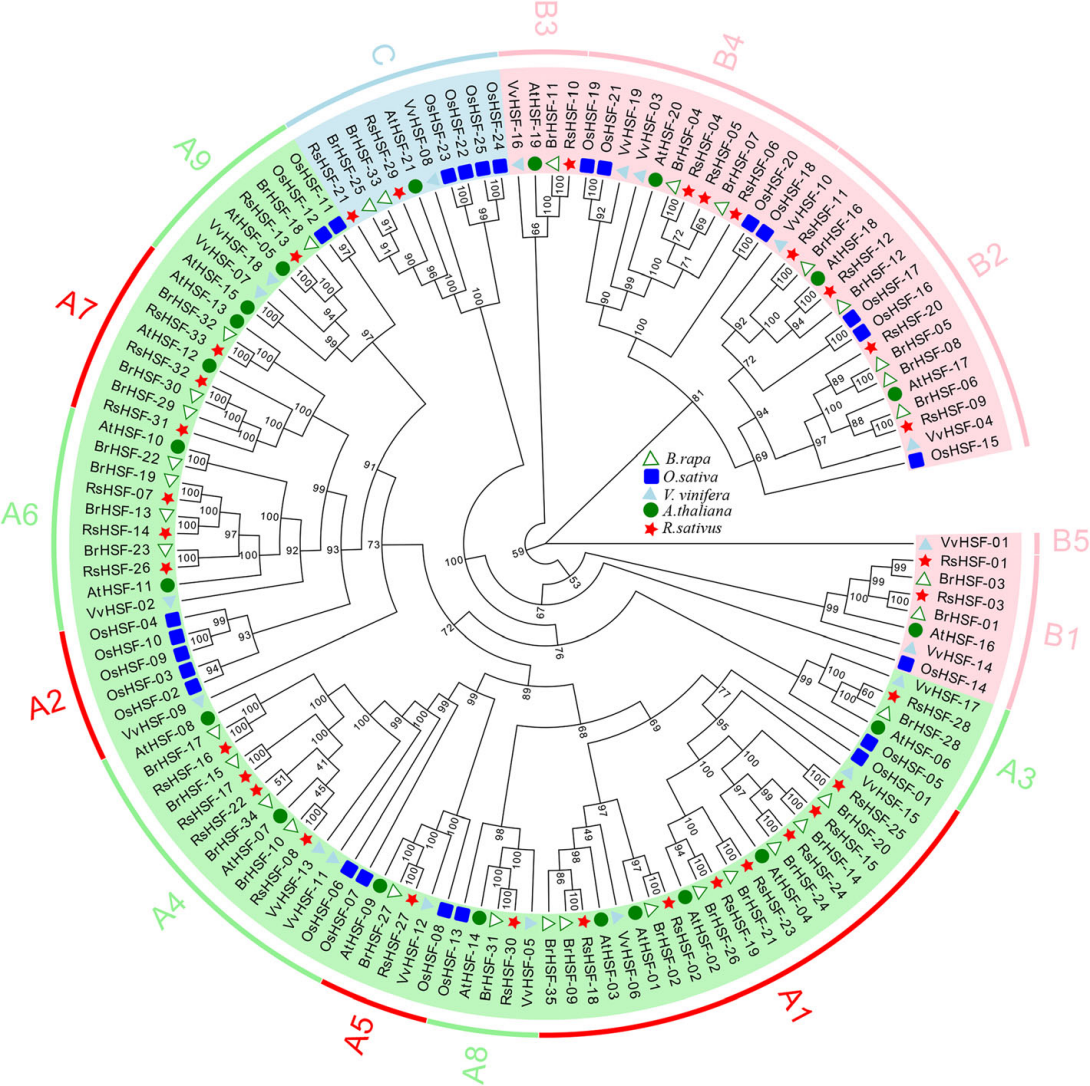
Phylogenetic tree constructed using the maximum likelihood method by IQ-TREE, with HSF proteins in radish, Chinese cabbage, *Arabidopsis*, grape and rice. Bootstrap values were generated using UFBoot with 1000 replicates and super-fast bootstraps were interpreted as a true node if the support was above 0.95

A phylogenetic tree was constructed using maximum likelihood methods by IQ-TREE [38] with the full sequences of the 133 HSF proteins consisted of 33 RsHSFs, 35 BrHSFs, 19 VvHSFs, 25 OsHSFs and 21 AtHSFs (Fig. 2). All proteins were categorised into three groups corresponding to the HSF classes A, B and C. Additionally, there were 9 RsHSF class A subgroups clearly obtained on the basis of the bootstrap values and phylogenetic analysis of *Arabidopsis* and rice. According to the phylogenetic tree, class A consisted of 8 subclasses (A1, A3, A4, A5, A6, A7, A8, and A9) and contained the most RsHSF numbers, while class C accounted for the least proportion of HSFs among these five species. Interestingly, no RsHSFs, AtHSFs or BrHSFs clustered into classes A2, whereas AcHSFs did not appear in classes A6 and A7. This finding suggests that most RsHSF genes are more closely related to their corresponding homologous genes in Chinese cabbage and *Arabidopsis*.

### Chromosomal location and duplication of RsHSF genes

**Fig. 3:**
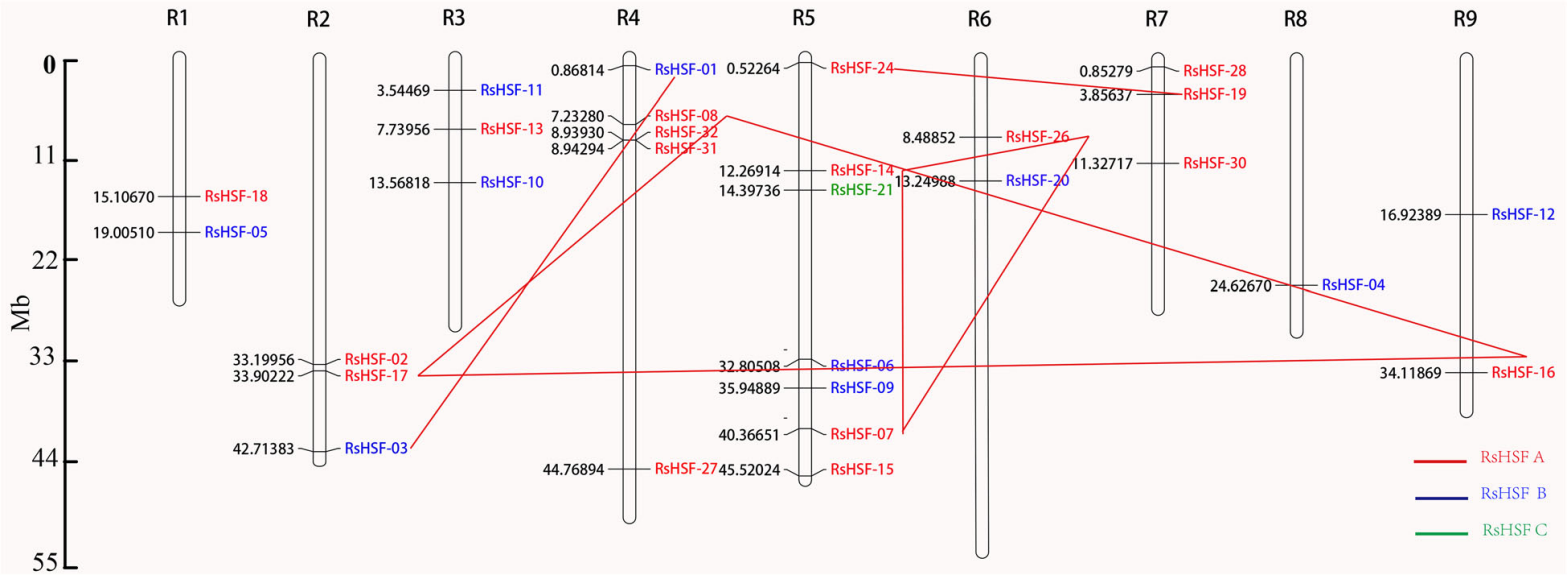
Distribution of RsHSF genes on nine chromosomes. Red, blue and green word represent class A, B and C, respectively. Red lines connect the RsHSF duplicated genes

To obtain the chromosome distributions of RsHSF genes, their DNA sequences were physically plotted on the chromosomes through blast searches against the genomic sequences (Additional file 5: Table S6). In total, 28 RsHSF genes were mapped on the chromosomes with uneven distribution, with exception of five genes (*RsHSF*-22, 23, 25, 29 and 33) (Fig. 3). Chromosome (Chr.) 5 had the largest number of RsHSF genes among three classes, followed by Chr. 4 (5 RsHSF genes from class A and B). Only one RsHSF gene was present in Chr. 8, while Chr. 1, 6 and 9 harbored two genes. Moreover, all the class B RsHSF genes were mapped on the chromosomes.

Genome duplications have been recognized and contributed to the expansion of gene family in plants [39, 40]. MCScanX was used to obtain information about origins of duplicate RsHSF genes in radish genome [41]. 13 (46.4%) RsHSF genes were duplicated and retained from a whole genome duplication (WGD) event and 11 (39.3%) were duplicated and retained from a singleton event (Additional file 6: Table S7). Eight WGD events of 10 duplicated RsHSF genes were identified and classified into four groups. Among these four groups, two harbored three genes (*RsHSF-07*, *RsHSF-14* and *RsHSF-26*; *RsHSF-08*, *RsHSF-16* and *RsHSF-17*), and these six genes were located on five chromosomes (Chr.2, 4, 5, 6 and 9). The other two groups contained two WGD events of four genes, and one duplication event harbored *RsHSF-01* and *RsHSF-03* which were located on Chr. 2 and 4, respectively (Fig. 3). Only one WGD event (*RsHSF-07*and *RsHSF-14*) took place within the same chromosome (Fig. 3, Additional file 7: Table S8). These results revealed that WGD or segmental duplication played an important role in the expansion of the radish HSF gene family.

### Identification of orthologous and paralogous HSF genes

**Fig. 4:**
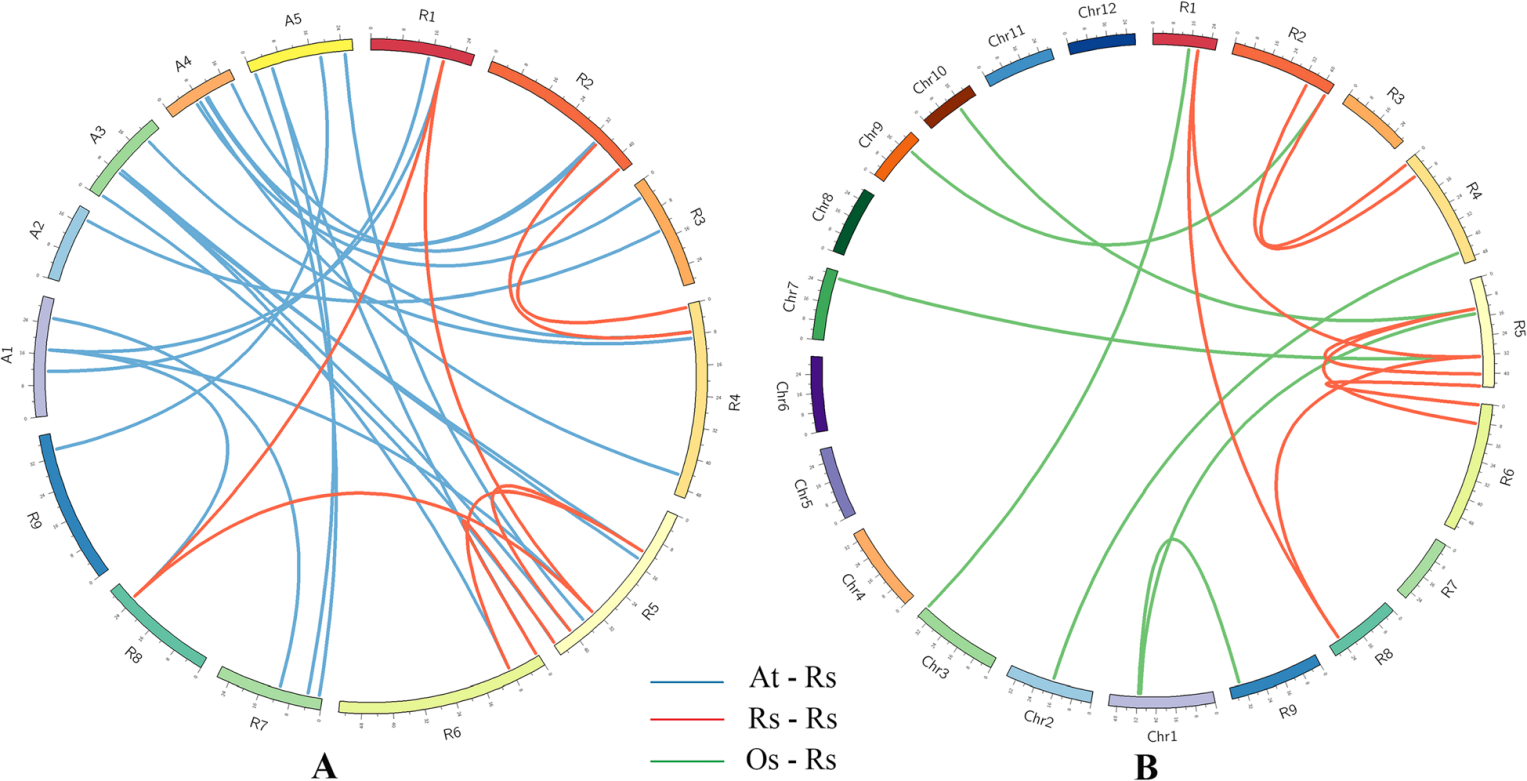
Orthologous and paralogous genes of HSF. **a** Radish (R01–R09) and five Arabidopsis chromosome (A1–A5) maps were based on orthologous and paralogous pair positions; **b** Radish and rice chromosome (Chr1–Chr12) maps were based on orthologous and paralogous pair positions with Circos

Polyploidization events contributed significantly to the evolution of flowering plants [42]. Previous studies showed that the common ancestor of *Brassica* and *Raphanus* have experienced α’ whole-genome triplication (WGT) event since its divergence from *Arabidopsis*. However, the gene losses of orthologous groups between *Arabidopsis*, *Brassica* and *Raphanus* for protein-coding genes occurred in both the *Brassica* and *Raphanus* lineages [39, 42]. Orthologous HSF genes among radish, *Arabidopsis* and rice were identified for triplication assessment using the Orthomcl-pipeline [43]. In total, there were 23 pairs of orthologous HSFs between radish and *Arabidopsis*, and eight paralogous gene pairs were identified in radish (Additional file 8: Table S9). It was reported that there are only six orthologous gene pairs between *Arabidopsis* and rice [23]. In addition, seven orthologous HSF gene pairs were identified between radish and rice (Additional file 8: Table S9). Among the orthologous HSF gene pairs between radish and *Arabidopsis*, *AtHSF-11* and *AtHSF-20* had three orthologous genes, three *AtHSFs* have two orthologous genes and 11 *AtHSFs* only had one orthologous gene (Additional file 2: Figure S3). These results indicate that many orthologous groups experienced gene losses. Moreover, five genes (*RsHSF-01*, *RsHSF-12*, *RsHSF-13*, *RsHSF-20* and *RsHSF-32*) had no orthologous gene in either *Arabidopsis* or rice. These results provide useful resource and reference for further exploring the functions of RsHSF genes in radish (Fig. 4).

### Expression pattern of RsHSF genes in different tissues

**Fig. 5:**
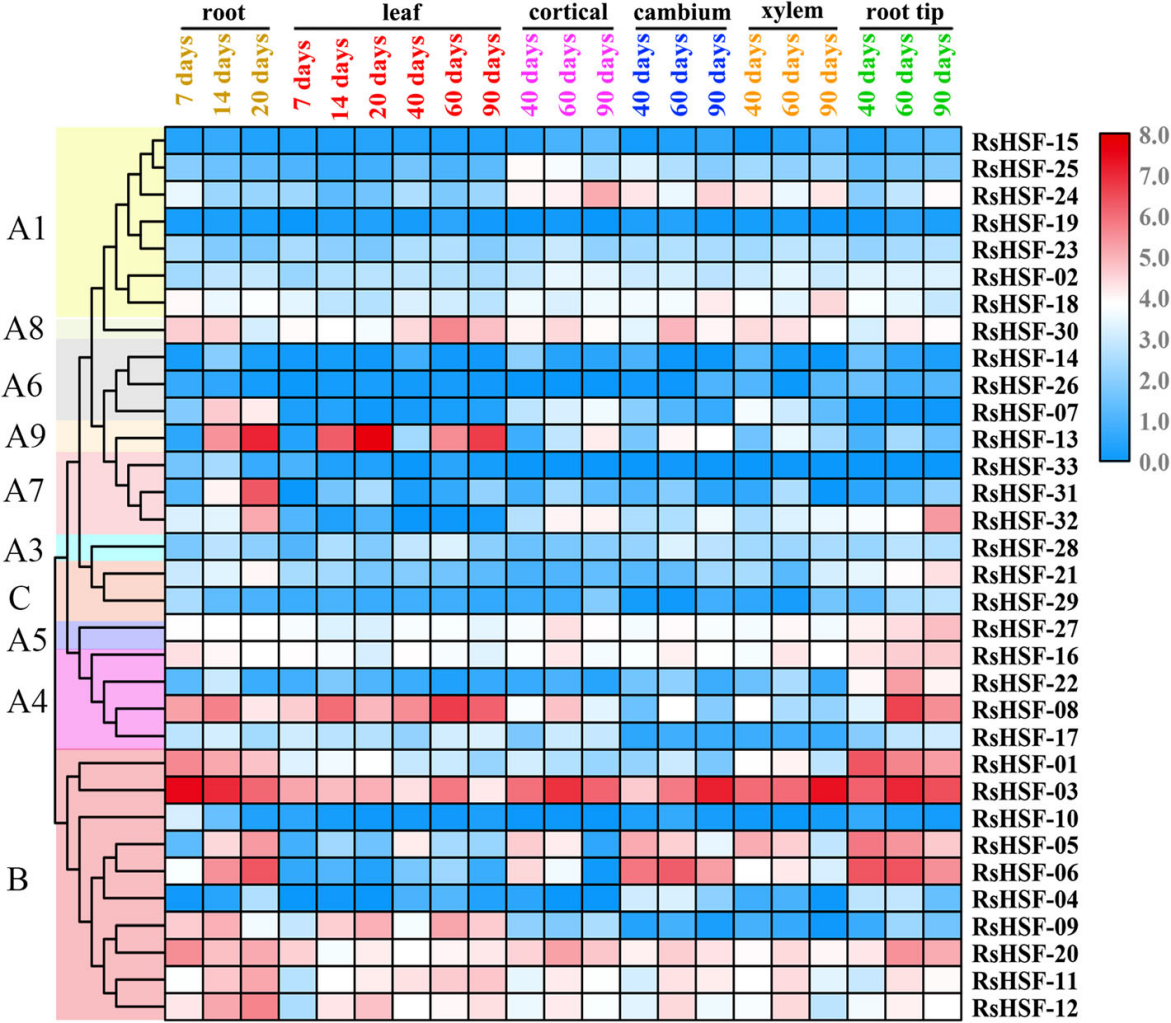
Heatmap of RsHSF genes in different stages and tissues. The color scale is based on the log_2_ (RPKM + 1) values

To determine spatiotemporal expression patterns of RsHSF genes, the Reads Per Kilobase Per Million (RPKM) values of the 33 RsHSF genes in different tissues and developmental stages were collected from RNA-Seq data and presented in a heatmap (Fig. 5, Additional file 9: Table S10). In general, a proportion of RsHSF genes were not expressed in several tissues and developmental stages. As shown in Fig. 5, the RPKM value varied from 0 to 127.5 among the 33 RsHSFs genes. The number of RsHSF genes with RPKM > 1 was 14 (42.4%). It was found that the RPKM values of four *RsHSFA1s* (*RsHSF-15*, *19*, *23*, *25*) were < 6 during all tissues and stages. Notably, among the other three *RsHSFA1* (*RsHSF-02*, *18*, *24*) genes, *RsHSF-18* and *RsHSF-24* were highly expressed in the cortex, cambium and xylem. The majority of RsHSF genes exhibited differential expression patterns in the various tissues and developmental stages. *RsHSF-21* and *RsHSF-29* (class C) were relatively lowly expressed during all developmental stages. In addition, *RsHSF-13* expression increased significantly in root after 7 days after sowing (DAS), and it was increased in leaves after 7 and 40 DAS as well. *RsHSF-30* (class A8) expression was higher compared to *RsHSF-28* (class A3) during all developmental stages. Besides, RsHSFB genes exhibited stage-specific expression patterns. Among class B, both *RsHSF-04* and *RsHSF-10* were lowly expressed during all stages, while *RsHSF-03* was highly expressed.

### RsHSF gene expression profile under abiotic stresses

**Fig. 6:**
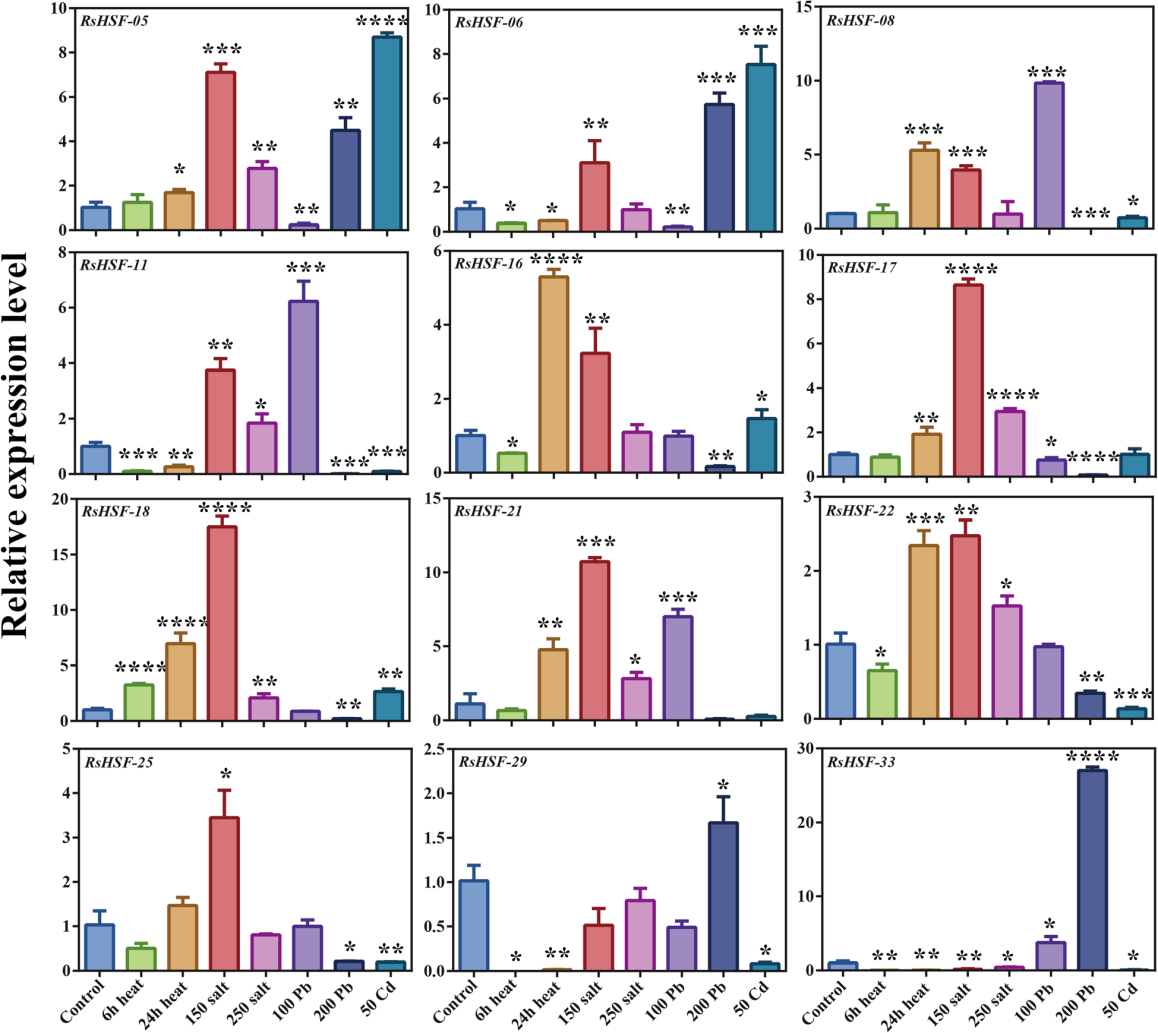
The expression levels of representative RsHSF genes under different stresses (cadmium, lead, heat, and salt stress). The subgroup is marked in different colors under the gene name. Each bar shows the mean ± SE of the triplicate assay. GraphPad Prism software was used to analysis and asterisks reveal the gene significantly upregulated or downregulated under abiotic stresses by t test (*P < 0.05, **P < 0.01, *** < 0.001)

RNA-Seq data showed that some HSF genes were differentially expressed under disparate stresses. The transcription levels (log_2_ (Fold Change) and log_2_TPM value) of certain RsHSF genes from the NCBI SRA were further analyzed (Additional file 2: Figure S4). Several HSF genes, including HSFA1s were up-regulated in response to heat and salt stresses. RsHSFA4s (*RsHSF-08*, *RsHSF-16* and *RsHSF-22*) were up-regulated under salt and lead (Pb) stresses, while they were down-regulated under HS. Additionally, HSFC1 (*RsHSF-21*) expression increased after Cd and salt treatments. HSFA1s as master regulators play vital roles in the HSR and activation of transcriptional networks. Expression levels of genes that encode HS responsive TFs including *DREB2A*, HSFA7s and HSFBs, are directly regulated by HSFA1s [44]. *HsfA4a* is induced by oxidative stress, including HS, and regulates *APX1* expression [45]. To gain insight into the expression patterns of HSF genes in radish, 12 RsHSF genes that belonged to different groups were verified by RT-qPCR analysis under heat, salt, Pb, and Cd stress (Additional file 10: Table S11). Overall, the gene expression patterns varied significantly under various treatments (Fig. 6). Most genes were up-regulated after 24 h HS exposure, while some genes were down-regulated after HS for 6 h (Fig. 6), such as *RsHSF-16*, *RsHSF-22* and *RsHSF-29*. Moreover, *RsHSF-18* expression was highly increased after 6 h HS, implying that it can quickly respond to this stress. In addition, *RsHSF-18* was up-regulated under Cd treatment and during all heat and salt stress time points, while *RsHSF-33* was only up-regulated in response to Pb stress. *RsHSF-05*, *RsHSF-06*, and *RsHSF-33* were up-regulated under 200 mg/L Pb treatment and are likely involved in the Pb stress response. *RsHSF-11* and *RsHSF-22*, exhibited relatively high expression level under salt stress, indicating that these two RsHSFs might play crucial roles in the biological process of salt stress response.

## Discussion

Radish (*Raphanus sativus* L.), an ancient cultivated crop worldwide, is an important human dietary component. Industrial development, climate change and increased areas of contaminated soil considerably affect the expansion and healthy cultivation of the radish. The HSFs family allows plants to cope with abiotic stresses (e.g. heat, Cd and high light) by regulating gene expression to prevent damage [15, 46]. Recently, the availability of increasingly complete genome sequencing has enabled the application of a bioinformatics approach to identify various gene families, including HSF in different species [27, 28]. However, the available information about the RsHSFs is limited. This study is the first comprehensive overview of the HSF gene family within the radish genome.

### Genome-wide identification of RsHSF genes

It is widely accepted that the HSF-type DBD and OD domains are necessary HSF structural components. The highly conserved DBD is composed of a three-helical bundle (H1, H2 and H3) and a four-stranded antiparallel ß-sheet [9]. The HR-A/B, connected to the DBD by a flexible linker of variable length (15–80 AAs), is comprised of hydrophobic heptad repeats [47, 48]. In brief, the DBD ensures HSFs combine with HSE, and the OD is the basis for differentiating the three HSF classes. It is reported that the DNA-binding domain of plant HSF is separated by a single intron. Although the position of this intron is unanimous in all HSF DBDs, the intron size is variable [21]. Therefore, we predicted the conserved domain using Heatster online tools [49] after performing Pfam and MARCOIL analysis. Consequently, 33 RsHSF genes were identified through genome-wide analysis (Additional file 1: Table S1–S3).

Among five plant species, 133 HSFs divided into three major classes were used to analyze the relationships. The results suggested that HSFs originated prior to the divergence of these species. Phylogenetic analysis evidenced that RsHSFs were more similar to BrHSFs and AtHSFs than OsHSFs, which corresponds with the fact that the radish, Chinese cabbage and *Arabidopsis* belong to Brassicaceae family (Fig. 2). The radish HSF number (33) was larger than that in *Arabidopsis* (21) and grape (19). Previous studies showed that distinct plants species harbor different numbers of HSFs, and land plants have more TFs than algae. These results indicate that the number of HSFs is probably related to the evolution and the growth environment [13].

### The evolutionary characterization of the RsHSF family

Gene duplication plays a major role in the evolution of novel gene functions and the expansion of gene families [36]. Among the evolutionary process in plants, gene duplication that increases the genome size and relaxes selection on one gene copy, is regarded as the primary driving force to allow the acquisition of new function, and enhance environmental adaptability [13, 50]. In Brassicaceae family, there were three WGDs after an *Arabidopsis* lineage diverged from the monocot lineage. The most recent WGD event occurred 50–65 million years ago, which was earlier than the divergence of plants in the Brassicaceae family [40, 51, 52]. Many gene families are expanded in higher plants, including *CML* [53], ALDHs [54], MADS-Box [55], NAC [56] and bHLH [57]. Current evidence shows that the evolution patterns of α’ duplicates are similar in *Raphanus* and *Brassica* [40]. For RsHSFs evolution, the expansion was due to eight duplicated pairs, which are consistent with those in Chinese cabbage.

Polyploidization events apparently occurred 3–12 million years after independence of the *Arabidopsis–Brassica* lineages, and the time of the *Raphanus* genus diverging from *Brassica* was longer than previously predicted [58, 59]. Furthermore, recent reports revealed that ~ 60% of genes that belong to the neopolyploid ancestor of *Raphanus* and *Brassica* disappeared due to a WGT event. However, a considerable number of genes still exist within the *Raphanus* and *Brassica* genomes, a phenomenon that represents the benefit of this evolutionary novelty [40]. Phylogenetic analysis of HSF genes among radish, *Arabidopsis* and Chinese cabbage indicated that many RsHSF genes are highly similar to their corresponding *Arabidopsis* and Chinese cabbage homologs. Moreover, a large number of the identified RsHSFs were detected as orthologous genes in *Arabidopsis*, including *RsHSF-04, RsHSF-05 and RsHSF-06*, which were the orthologous genes of *AtHSF-20* (Fig. 4). These results provide valuable cues on further understanding the functions of these highly homologous genes in radish. For instance, *RsHSF-07*, *RsHSF-14* and *RsHSF-26* were highly similar to *AtHSF-11*, which is vital for HS tolerance and is a downstream regulator during the ABA-mediated stress response [15]. In this study, we identified eight pairs of RsHSF paralogs that might be related to the extended genome triplication of the radish. Collectively, the RsHSF gene family expansion may be largely related with gene duplication, and the identification of the orthologous gene pairs between radish and *Arabidopsis* provided a reference for exploring the roles of HSFs under different stresses in radish.

### The potential roles of differentially expressed RsHSF genes

HSFs play important roles in plant response to abiotic stresses by regulating the expression of different genes [13, 23, 60]. Among HSF genes in plants, *HsfA1* (a major transcriptional activator) is necessary to evoke the HSR. Indeed, the *HsfA1*-knockout mutant shows reduced expression of many HS-induced genes [45, 61]. Moreover, *HsfA1a* is associated with Cd tolerance by improving melatonin biosynthesis [8]. In this study, RsHSF genes were significantly differentially expressed in response to various stresses. RsHSFA1s (*RsHSF-18* and *RsHSF-25*) showed converse expression levels upon 24 h HS or 200 mg/L Pb, which was significantly down-regulated under Pb stress, indicating that the expression of *HSFA1* may be repressed under 200 mg/L Pb stress. In addition, *RsHSF-18* and *RsHSF-25* were differentially expressed under 50 mg/L Cd stress, which may be related to the specific function in the Cd stress response. *HsfA4A* enhances Cd tolerance in wheat and rice and salt protection in *Arabidopsis* [62]. RsHSFA4s (*RsHSF-08*, *RsHSF-16*, *RsHSF-17* and *RsHSF-22*) were up-regulated, while only *RsHSF-16* was highly expressed upon salt and Cd treatment. These up-regulated genes might play specific roles in coping with stresses in radish plants. Unlike HSFAs, a considerable number of HSFBs and HSFCs are not reported to act as transcription activators, although tomato HSFB1 apparently plays a role as a transcriptional co-activator of HsfA1 under HS [17, 44, 63]. In this study, the expression of RsHSFBs was distinct. For example, *RsHSF-08* and *RsHSF-18* had high expression level while *RsHSF-11* and *RsHSF-33* were down-regulated upon 24 h HS. Intriguingly, the expression level of *RsHSF-29* (an *HSFC*), was down-regulated under all abiotic stresses except 200 mg/L Pb treatment. *HSFA1d* and *HSFA3* were significantly up-regulated in radish under HS [30], while several *HSFs*, including *HSFC1* and *HSFB2a*, were notably up-regulated under salt stress [64]. Some heavy metal stress responsive signaling molecules are activated on detecting Cd^2+^, such as calcium-dependent protein kinases (CDPKs) and mitogen-activated protein kinase (MAPKs), both of which consequently regulate various metal-responsive TF families (eg., NACs and HSFs) in radish nucleus [65]. Moreover, four radish HSF genes were up-regulated under Cr stress in radish [66]. Taken together, several RsHSF genes were differentially expressed upon abiotic stresses including heat, salt or heavy metal stress, and these results indicate that they might be involved in the plant response to abiotic stresses.

RsHSF gene expression during different stages and in tissues is associated with abiotic stresses tolerance. In this study, RsHSF genes exhibited tissue-specific expression patterns. At the seedling stage, *RsHSF-04* and *RsHSF-33* were lowly expressed in 20 and 14 DAS root, respectively. Throughout development stages, *RsHSF-10* was only detected in 7 and 14 DAS root. *AtHSF-16* (At4g36990) is a direct target gene of AtHsfA1s, which are critical for plants to initiate positive activities when coping with HS [16, 67]. *RsHSF-03* is highly similar to *AtHSF-16* (At4g36990) and exhibited high expression level during seedling and taproot-thickening stages. Notably, *RsHSF-19*, the orthologous gene of *AtHSF-02*(At5g1682), was not expressed during all stages. These findings imply that the highly expressed genes among different stages and tissues may contribute to enhance stress tolerance in radish. Further characterization of these differentially expressed RsHSF genes would facilitate investigating the regulatory networks of abiotic stress responses in radish.

## Conclusions

In summary, a total of 33 RsHSF genes were identified at the genome-wide level in radish. The protein motifs in most HSF members within one class were similar, and most HSF genes had only one intron. The expression patterns of several RsHSF genes were more similar within the same class than that among different classes. Moreover, comparative analysis revealed a series of paralogous RsHSF genes in radish and orthologous RsHSF genes in *Arabidopsis* and rice. These results may enhance our understanding of RsHSF functions and their involvement in the radish stress responses. Identification of RsHSF genes provides a rich resource for comprehensive investigation of the roles of HSF genes in regulatory networks of abiotic stress responses in plants. These findings will facilitate further functional characterization of RsHSF genes, and provide valuable information to clarify the molecular mechanism underlying abiotic stress responses in radish.

## Methods

### Sequence collection and HSF identification

Whole genome sequences of the radish HSF family were identified from the NODAI Radish genome database [31]. The HSF family sequences identified from *Arabidopsis* and Chinese cabbage were downloaded from the *Arabidopsis* Information Resource [37] and *Brassica* database (BRAD) [33], respectively. The HSF family members for rice (*O. sativa*) and grape (*V. vinifera*) were obtained from the Rice Genome Annotation Project [36] and Grape Genome Browser (http://www.genoscope.cns.fr/externe/GenomeBrowser/Vitis/) [34], respectively.

To confirm the radish HSF family candidates, proteins with the HSF-type DBD domain (Pfam accession number: PF00447) were searched against the genome protein sequences using the HMM search tool with an E-value cut-off of 0.01 [32].

### Phylogenetic analysis and characterization of RsHSFs

RsHSF DBD and oligomerization (HR-A/B or OD) domains were detected using SMART (http://smart.embl-heidelberg.de/) [68], Pfam [69], MARCOIL (http://toolkit.tuebingen.mpg.de/marcoil) and Heatster online tools (http://www.cibiv.at/services/hsf/info) [49]. NLS and NES domains were predicted using NLStradamus (http://www.moseslab.csb.utoronto.ca/NLStradamus/) and NESs (http://www.cbs.dtu.dk/services/NetNES/), respectively. RsHSF protein properties including molecular weight, theoretical pI and instability index were analyzed with the ExPASy ProtParam tool (https://www.expasy.org/). The alignment was firstly performed using MUSCLE and consequently the best-fit model was determined by MEGA X. The phylogenetic tree was constructed with specific parameters (model: JTT + G) by IQ-TREE (1000 ultrafast bootstraps) using the maximum likelihood method [38]. The phylogenetic tree was displayed using EVOLVIEW (http://www.evolgenius.info/evolview/).

### Gene structure and conserved motif analysis

RsHSF gene exton/intron organization was obtained from GSDS (http://gsds.cbi.pku.edu.cn/) [70] by aligning coding sequences with their corresponding genomic DNA sequences. The conserved motifs within the determined HSF groups were identified by MEME (version4.11.1) (http://meme-suite.org/) based on the following parameters: number of repetitions, any; maximum number of motifs, 25; and the optimum motif widths, 6–50 AAs [71]. The conserved motifs were visualized by Tbtools [72].

### Chromosomal localization and identification of orthologous and paralogous *HSFs*

RsHSF gene sequences in scaffolds were obtained from the NODAI Radish Genome Database [31], and local BLAST was performed against Radish Genome Database [73]. Gene sequences with ≥98% identity and length difference ≤ 5 base pairs were considered to be the same genes between two genomes, and the corresponding location of RsHSF genes in chromosomes was localized using MapChart Software [74]. The Multiple Collinearity Scan toolkit (MCScanX) was used to identify the RsHSF duplication events [41, 73]. BLASTP was performed to identify the intra-species paralogous pairs using protein sequences with the following parameters settings: 1) alignment significance: E_VALUE (default: 1e-05); 2) MATCH_SCORE: final score (default: 50); 3) MATCH_SIZE: number of genes required to call a collinear block (default: 5) and the maximum gaps (default: 25).

The OrthoMCL pipeline [43] was used with standard settings to identify potential orthologous and paralogous HSF genes in radish, *Arabidopsis*, and rice. The relationships of orthologous and paralogous genes among the three species were plotted using Circos software [75].

### Expression analyses of RsHSF genes by RNA-Seq

To analyze the expression patterns of the RsHSF genes, the Illumina RNA-Seq data from five tissues (cortex, cambium, xylem, root tip and leaf) and six stages [7, 14, 20, 40, 60 and 90 days after sowing (DAS)] were collected from the radish reference genome (http://www.nodai-genome-d.org/) [31] (Additional file 11: Table S12). The expression level for each RsHSF gene was presented with the RPKM method and heat map was generated using TBtools [72].

### Abiotic stress treatments

‘NAU-YH’ seeds, a radish advanced inbred line with small red global roots, were sterilised, rinsed and incubated for 3 days. Germinated seeds were transferred into a plug tray and cultured in a growth chamber at 25 °C day/18 °C night with 14 h light/10 h dark, 60% humidity and 12,000 lx light. Three weeks later, seedlings with 6–7 true leaves at the cortex split stage were exposed to 38 °C for 0, 6 or 24 h (termed control, Heat6 or Heat24, respectively). For salt and heavy metal (Cd and Pb) treatment, seedlings of identical sizes were grown in a plastic container with modified half-strength Hoagland nutrient solution as described previously [76]. The nutrient solution was changed every 3 days. One week later, the seedlings were treated with CdCl_2_⋅2.5H_2_O (50 mg/L), Pb(NO_3_)_2_ (100 mg/L or 200 mg/L) and NaCl (150 mM or 250 mM), respectively [29]. Seedlings grown in Cd/Pb-free nutrient solution were used as the control. In this study, three similar seedlings with 6 true leaves at the cortex split stage were used for each treatment, and each treatment was performed in triplicate. Additionally, 0.1 g root tissue was used for RNA isolation from each sample. These samples were immediately frozen in liquid nitrogen and stored at − 80 °C for further analysis.

### RNA-Seq data and RT-qPCR analysis

In this study, the raw RNA-Seq data was collected from NCBI Sequence Read Archive (SRA, http://www.ncbi.nlm.nih.gov/sra) using the SRA Toolkit (ver.2.8.2). After removing the adaptor sequences, contaminants and low-quality reads, the clean reads were mapped to the radish reference genome using TopHat2 [77]. Transcripts per Kilobase Million (TPM) values were estimated using Salmon [78] for different sample comparisons. Differential gene expression and the adjusted *p* values were calculated using the edgeR package [79].

Total RNA was isolated from the control and treated radish roots using RNAsimple total RNA kit (Tiangen, Beijing, China).Then, the RNA was reverse transcribed into cDNA uisng PrimeScript™ II 1st Strand cDNA Synthesis Kit (Takara, Dalian, China) according to the manufacturer’s instructions. RT-qPCR analysis was conducted using SYBR Green PCR Master Mix (Takara, Dalian, China) [29]. *RsActin* was employed as the internal standard to normalize expression [80]. Each 20 μl reaction contained 10 μl of 2 × SYBR Green PCR Master Mix (Takara, Dalian, China), 0.2 μM of each primer and 2 μl diluted cDNA. PCR was performed on an iCycler iQ real-time PCR detection system (BIO-RAD, USA) with the following thermal cycling conditions: 95 °C for 3 min, and 40 cycles of 95 °C for 5 s, 58 °C for 30 s, and 72 °C for 10 s. The 2^-ΔΔ*C*^_T_ method was used to calculate relative expression level [81]. Three biological and technical replicates were performed. The significance of differences between groups was evaluated using a Student’s t test. Analyses were performed with GraphPad Prism software (GraphPad Software, San Diego, California).

## Supplementary information

**Additional file 1: Table S1.** Amino acid sequences of the HSF proteins from NODAI Radish genome database. **Table S2.** CDS sequences of the *HSF* genes from NODAI Radish genome database. **Table S3.** DNA sequences of the *HSF* genes from NODAI Radish genome database.

**Additional file 2: Figure S1** Protein sequence alignment of the DBD domain identified in all RsHsf genes. **Figure S2.** The LOGO of 25 amino acid motifs in HSF proteins. **Figure S3.** The networks of HSF genes in radish and *Arabidopsis*. This interrelation network has been constructed using radish and *Arabidopsis* orthologous gene pairs. **Figure S4.** The differential expression profiles of some HSF genes in radish under the Heat, salt, Pb and Cd treatments. The color scale of heatmap is based on the log_2_Foldchange (a) and log_2_TPM value (b), respectively.

**Additional file 3: Table S4.** Summary information of the RsHSF proteins.

**Additional file 4: Table S5.** HSFs in Arabidopsis, rice, and grape.

**Additional file 5: Table S6.** Summary information of chromosomal location of RsHSFs in radish.

**Additional file 6: Table S7.** The different origins of HSF genes in radish.

**Additional file 7: Table S8.** Duplicated genes of *HSF* in radish.

**Additional file 8: Table S9.** The orthologous and paralogous gene pairs of HSF proteins among the radish, Arabidopsis and rice.

**Additional file 9: Table S10.** Tissue-specific expression of radish HSF genes.

**Additional file 10: Table S11.** Relative expression of RsHSF genes under different treatments determined by RT-qPCR.

**Additional file 11: Table S12.** The accession number of RNAseq data.

## Data Availability

The datasets supporting the conclusions of this article are included within the article and its additional files (Additional file 11: Table S12).

## Abbreviations

CDS: Coding sequence
DAS: Days after sowing
DBD: DNA-binding domain
DEGs: Differentially expressed genes
HMM: Hidden Markov Model
HR-A/B: Hydrophobic amino acid residues
HSF: Heat shock transcription factor
HSPs: Heat shock proteins
HSR: Heat stress response
MWs: Molecular weights
NES: Nuclear export signal
NLS: Nuclear localization Signal
OD: Oligomerization domain
pI: Theoretical isoelectric points
RT-qPCR: Reverse-transcription quantitative PCR
TDR: Temperature-dependent repression
TF: Transcription factor
WGD: Whole-genome duplication
WGT: Whole genome triplication
