# Supplementary material for: Genome-wide characterization and evolutionary analysis of heat shock transcription factors (HSFs) to reveal their potential role under abiotic stresses in radish (Raphanus sativus L.)
Source: BMC Genomics. 2019 Oct 24;20:772. doi: 10.1186/s12864-019-6121-3 (PMC6814140; doi:10.1186/s12864-019-6121-3)
Supplement: Supplementary file 2 — Additional file 2: Figure S1 Protein sequence alignment of the DBD domain identified in all RsHsf genes. Figure S2. The LOGO of 25 amino acid motifs in HSF proteins. Figure S3. The networks of HSF genes in radish and Arabidopsis. This interrelation network has been constructed using radish and Arabidopsis orthologous gene pairs. Figure S4. The differential expression profiles of some HSF genes in radish under the Heat, salt, Pb and Cd treatments. The color scale of heatmap is based on the log2Foldchange (a) and log2TPM value (b), respectively. [file 12864_2019_6121_MOESM2_ESM.doc]

**Figure.S1** Protein sequence alignment of the DBD domain identified in for all RsHsf genes.

**
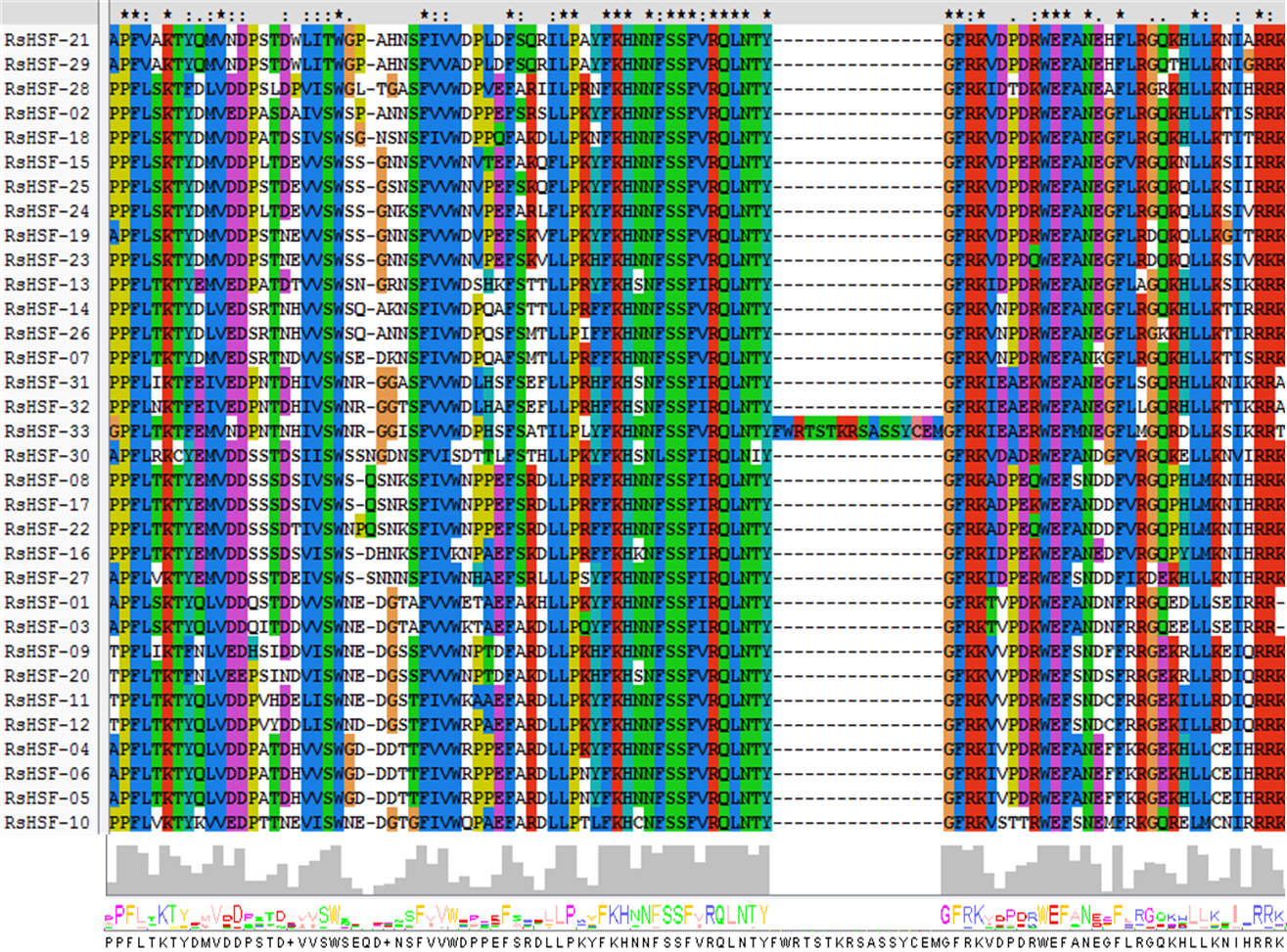
**

**Figure.S2** The LOGO of 25 amino acid motifs in HSF proteins


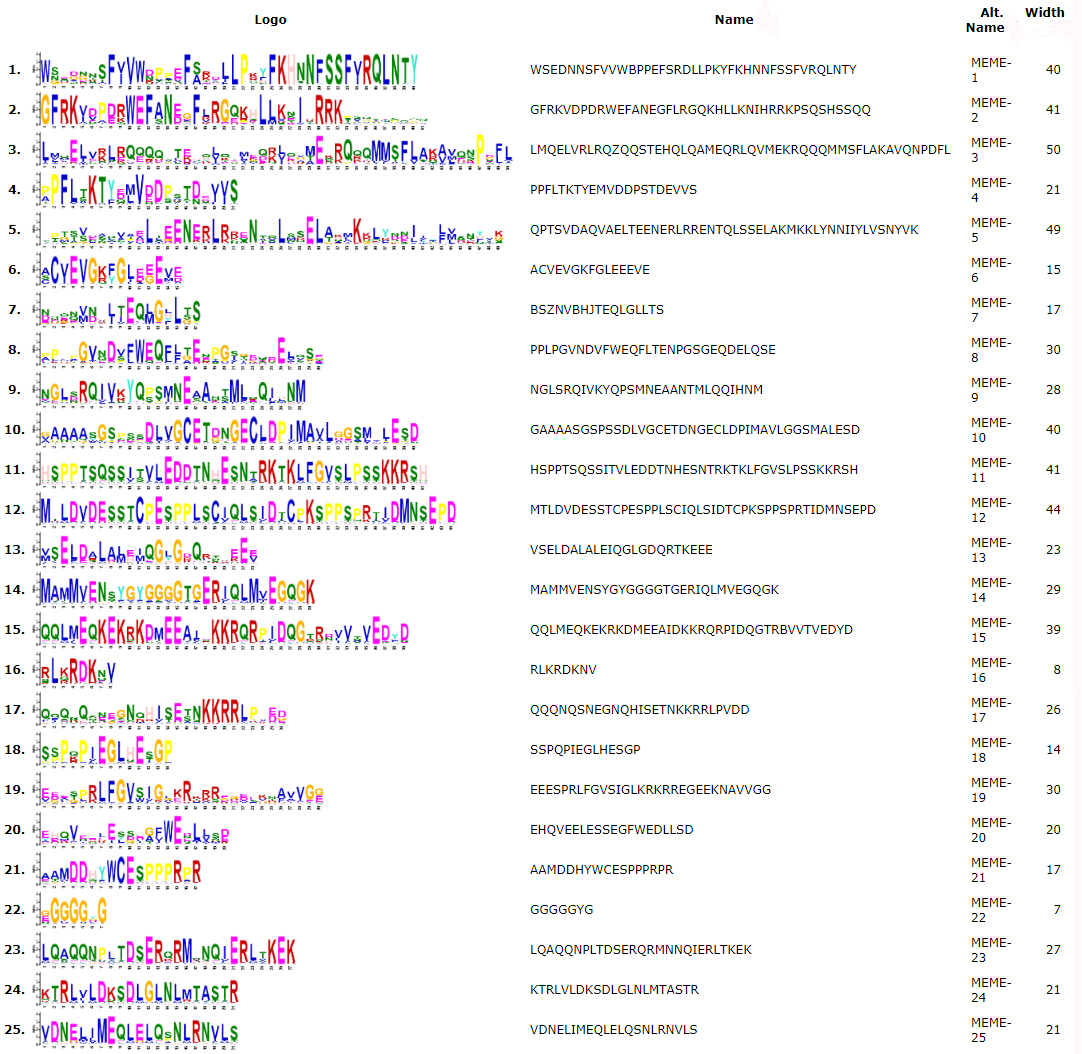


**Figure.S3** The networks of HSF genes in radish and *Arabidopsis*. This interrelation network has been constructed using radish and *Arabidopsis* orthologous gene pairs


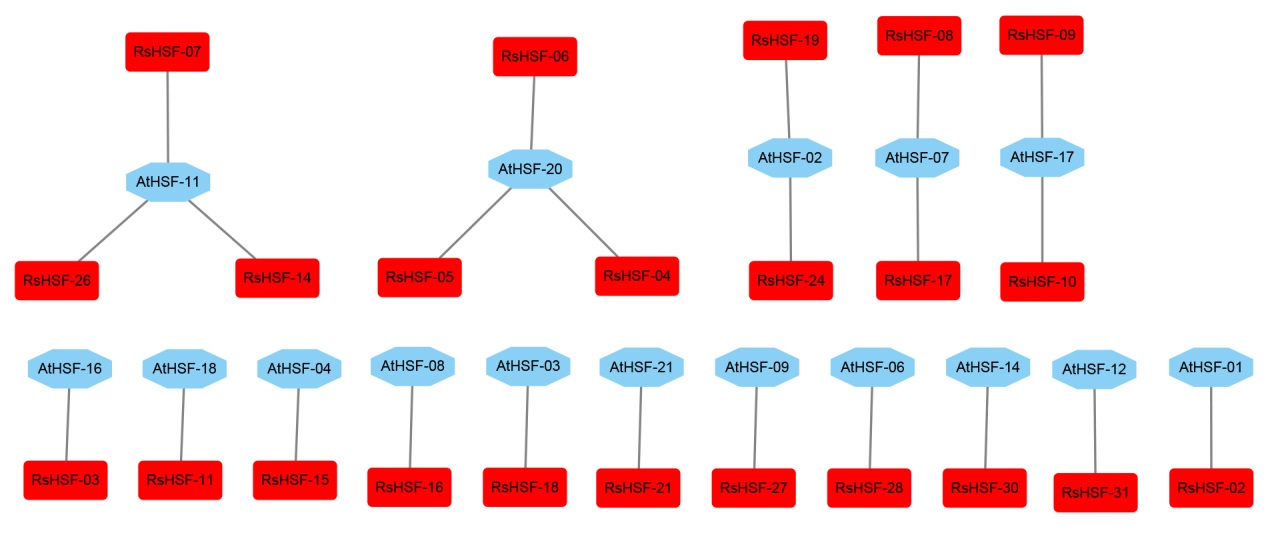


**Figure.S4** The differential expression profiles of some HSF genes in radish under the Heat, salt, Pb and Cd treatments in radish. The color scale of heatmap is based on the log_2_Foldchange (a) and log_2_TPM value (b), respectively.

**
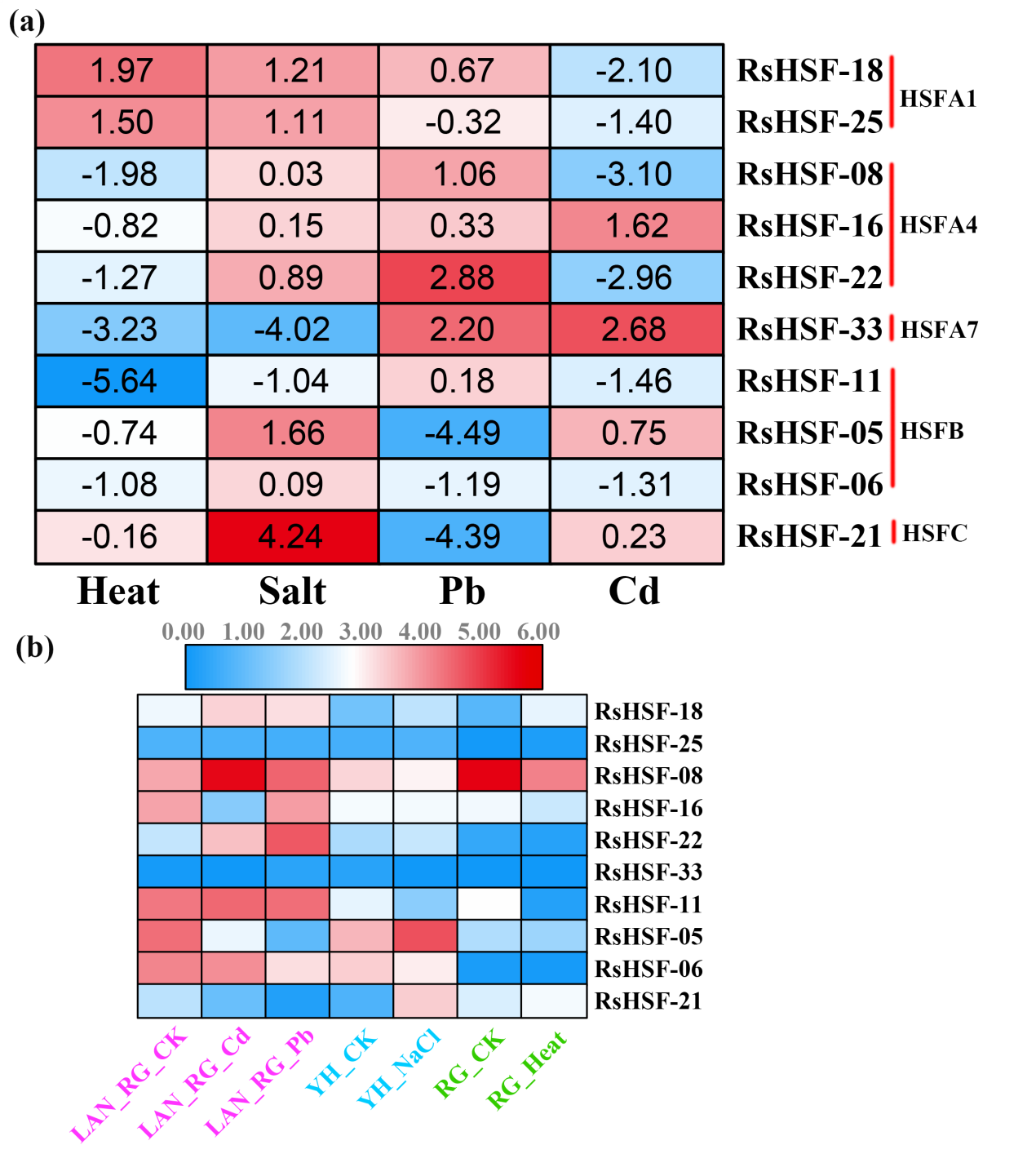
**
